# Modeling and Identification of the Rate-Dependent Hysteresis of Piezoelectric Actuator Using a Modified Prandtl-Ishlinskii Model

**DOI:** 10.3390/mi8040114

**Published:** 2017-04-04

**Authors:** Yanding Qin, Xin Zhao, Lu Zhou

**Affiliations:** 1 Institute of Robotics and Automatic Information System, Nankai University, Tianjin 300350, China; zhaoxin@nankai.edu.cn (X.Z.); zhoulu@nankai.edu.cn (L.Z.); 2 Tianjin Key Laboratory of Intelligent Robotics, Nankai University, Tianjin 300350, China

**Keywords:** piezoelectric actuator, hysteresis, rate-dependence, Prandtl-Ishlinskii

## Abstract

Piezoelectric actuator (PEA) is an ideal microscale and nanoscale actuator because of its ultra-precision positioning resolution. However, the inherent hysteretic nonlinearity significantly degrades the PEA’s accuracy. The measured hysteresis of PEA exhibits strong rate-dependence and saturation phenomena, increasing the difficulty in the hysteresis modeling and identification. In this paper, a modified Prandtl-Ishlinskii (PI) hysteresis model is proposed. The weights of the backlash operators are updated according to the input rates so as to account for the rate-dependence property. Subsequently, the saturation property is realized by cascading a polynomial operator with only odd powers. In order to improve the efficiency of the parameter identification, a special control input consisting of a superimposition of multiple sinusoidal signals is utilized. Because the input rate of such a control input covers a wide range, all the parameters of the hysteresis model can be identified through only one set of experimental data, and no additional curve-fitting is required. The effectiveness of the hysteresis modeling and identification methodology is verified on a PEA-driven flexure mechanism. Experimental results show that the modeling accuracy is on the same order of the noise level of the overall system.

## 1. Introduction

Piezoelectric actuator (PEA) has been widely utilized in ultra-precision positioning and manipulation applications due to its sub-nano motion resolution, high output force and fast response capabilities [1]. However, the disadvantages of the PEA are also distinct: (1) the PEA can be easily damaged by large bending torques or external impacts as the material is brittle; (2) the stroke of the PEA is very limited. The ratio between the stroke and the length of the PEA is typically on the level of 10 μm/cm; and (3) the inherent rate-dependent hysteretic and creeping nonlinearities significantly degrade the PEA’s motion accuracy. In practice, flexure-based displacement amplification mechanisms are generally adopted to magnify the stroke of the PEA, such as the flexural lever mechanism and the flexural Scott-Russell mechanism [2,3]. Capacity-based, laser-based sensors and strain gauges are generally utilized to measure such small displacements. For the motion control of PEAs, the hysteresis can be compensated using either the modeling-inversion based approaches [4,5,6] or the model-free feedback control [7]. Unlike the hysteresis, the creep is the slow drift of the PEA’s output over time that can be easily compensated through the feedback control.

Hysteresis modeling and compensation have been extensively investigated in recent decades. One widely employed hysteresis model is the Preisach model [8,9,10] which describes the hysteresis phenomenon through a double integral. The Preisach model can be used for hysteresis compensation and very high motion accuracy can be achieved if it is combined with other feedback controllers [11]. As the modeling accuracy of the Preisach model is highly related to the segmentation of the α-β plane, one needs to increase the model’s order to obtain higher modeling accuracy. Another widely employed hysteresis model is the Prandtl-Ishlinskii (PI) model [12,13]. The PI model is becoming more and more popular due to its simplicity in formulation, high modeling accuracy, and theoretical reversibility in the rate-independent form, making it attractive in real-time implementations. It must be noted that the classical PI model is static and symmetrical about the loop center. However, the measured hysteresis of the PEA exhibits strong rate-dependence and asymmetry (saturation) properties. Rate-dependence is the phenomenon in which the measured hysteresis curve of a PEA will become wider with the increment of the input rate. And the measured hysteresis curve of a PEA is not strictly symmetrical about its loop center, which is defined as the saturation property. These factors significantly increase the difficulty in hysteresis modeling and compensation. In literature, different modifications have been made to the classical PI model to better fit the saturation and rate-dependence properties of the measured hysteresis of PEAs [12,14,15,16].

The strong couplings between the hysteretic and creeping nonlinearities and the linear dynamics of a PEA make it impossible to isolate the hysteretic nonlinearity from its linear dynamics. Further, the output of a PEA is also susceptible to many factors, such as the preload force, the external load, and the dynamics of the transmission chains. This makes it difficult to precisely predict the behavior of a PEA if only a hysteresis model or a dynamics model is constructed. Therefore, an integrated model of both the linear dynamics and the nonlinear hysteresis will significantly improve the modeling accuracy of a PEA. In the research work of Hassani and Tjahjowidodo [17], both the dynamics of the mechanism and the hysteresis of the PEA are modeled and integrated together as a full hysteresis-dynamics model, where the hysteretic response of the PEA is adopted as the input to the linear dynamics of the mechanism. The combination of both the linear dynamics and nonlinear hysteresis obviously increases the modeling accuracy of the overall system.

The PEA’s dynamics is very important in the scanning- or vibration-based applications where the PEA moves very fast, such as the atomic force microscope and ultrasonic motor. However, in many micro and nano scale manipulations, such as in the manipulation and characterization of living cells [18,19], the endeffector follows the motion of the master operator’s hand, or moves very slowly, typically on the order of several Hertz. For these very slow motions, the PEA’s dynamics is not obvious and the PEA’s hysteresis becomes the dominant factor affecting the behavior of the overall system. Therefore, the hysteresis modeling and compensation is important to improve the performance of such systems. This paper focuses on the hysteresis modeling and identification of such systems. In our previous work [20], the saturation property was accounted for by the use of a polynomial operator, and the rate-dependence property was accounted for by varying the weight vector of the PI model according to the input rate. Although very high modeling accuracy was achieved, the threshold vector was still manually assigned. As a result, a trial and error process was inevitable, and a high level of knowledge on the characteristics of the PEA’s hysteresis was required. From the practitioner’s point of view, it is desirable to eliminate such a complex modeling and identification process to achieve ease of use in real implementations. In order to improve the applicability of the hysteresis modeling and compensation method proposed in our previous work [20], this paper aims to eliminate all the manual interventions during the parameter identification process. As a result, one only has to check the bounds of the input range from the manual of the PEA and select the order of the hysteresis model, and no other post processing or manual intervention is required during the parameter identification process. 

## 2. Materials and Methods 

### 2.1. Materials

**Figure 1:**
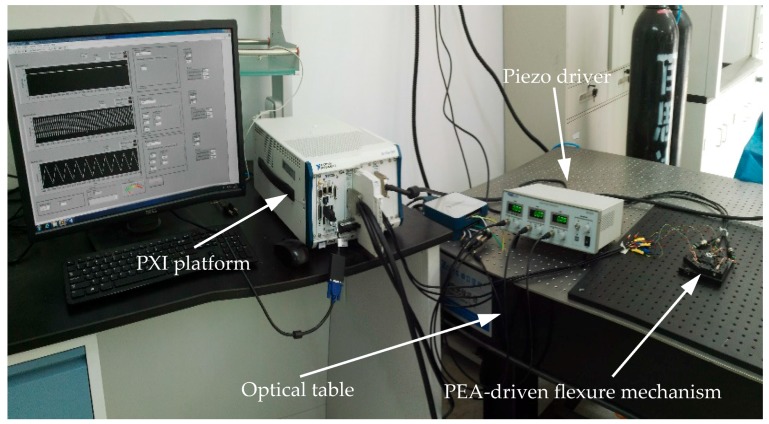
The experimental setup of the overall system.

A three degrees-of-freedom (DOF) flexure mechanism presented in [21] is utilized for the hysteresis modeling and verification in this paper. This mechanism is actuated by three PEAs (Model PZS001 from Thorlabs (Newton, NJ, USA)). The input range of the PEA is 0–10 V and the maximum displacement of the PEA is 11.6 ± 2.0 μm. The displacement of the PEA is measured by the strain gauges attached on the PEA in a full Wheatstone bridge configuration. The control voltages are exerted on the PEA through a piezo driver (Model MDT693B from Thorlabs). The data acquisition task is implemented on a PXI platform (Model 1082 equipped with a PXI-8135 controller, a PXIe-6363 data acquisition card and a TB-4330 bridge amplifier, all from National Instruments (Austin, TE, USA)) and runs in the real-time environment of Labview (Version 2014 SP1, National Instruments). As shown in Figure 1, the overall system is mounted on an optical table to isolate the ground disturbances. The noise level of the system is measured to be 100 nm. For the parameter identification and validation in this paper, different control signals are exerted on the PEA in one axis of the mechanism and the resultant extension of the PEA is measured by the strain gauges. 

### 2.2. Modified Prandtl-Ishlinskii Hysteresis Model

#### 2.2.1. Classical Prandtl-Ishlinskii Model

The basic component of all PI-based hysteresis models is the backlash operator in the following formulation:

(1) $$\begin{array}{l} H_{r}(u,t)=\max\{x(t)-r,\min\{u(t)+r,H_{r}(t-T)\}\}\\ H_{r}(u,0)=\max\{u(0)-r,\min\{u(0)+r,0\}\} \end{array}$$

where *H_r_*(*u*,*t*) denotes the backlash operator, *u*(*t*) and *y*(*t*) represent the input and output of the backlash operator, respectively, *r* is the threshold of the backlash operator, *t* is the current time, and the system runs with a sampling period of *T*. The initial condition can be set to zero as a PEA is typically activated from its de-energized state.

The classical PI model is defined as the weighted superposition of *n* backlash operators, i.e.,

(2) $$z(t)=\sum_{i=1}^{n}w_{i}H_{ri}(u,t)=[w_{1},w_{2},...,w_{n}]\cdot {\left[H_{r1}(u,t),H_{r2}(u,t),...,H_{rn}(u,t)\right]}^{T}=w^{T}\cdot H_{r}(u,t)$$

where *z*(*t*) is the output of the classical PI model, *n* is defined as the order of the PI model, **w** = [*w*_1_, *w*_2_, …, *w_n_*]*^T^* and **H_r_**(*u*,*t*) = [*H_r_*_1_(*u*,*t*), *H_r_*_2_(*u*,*t*), …, *H_rn_*(*u*,*t*)]*^T^* are the weight vector and the backlash operator vector, respectively. 

#### 2.2.2. Modeling of the Saturation Property

**Figure 2:**
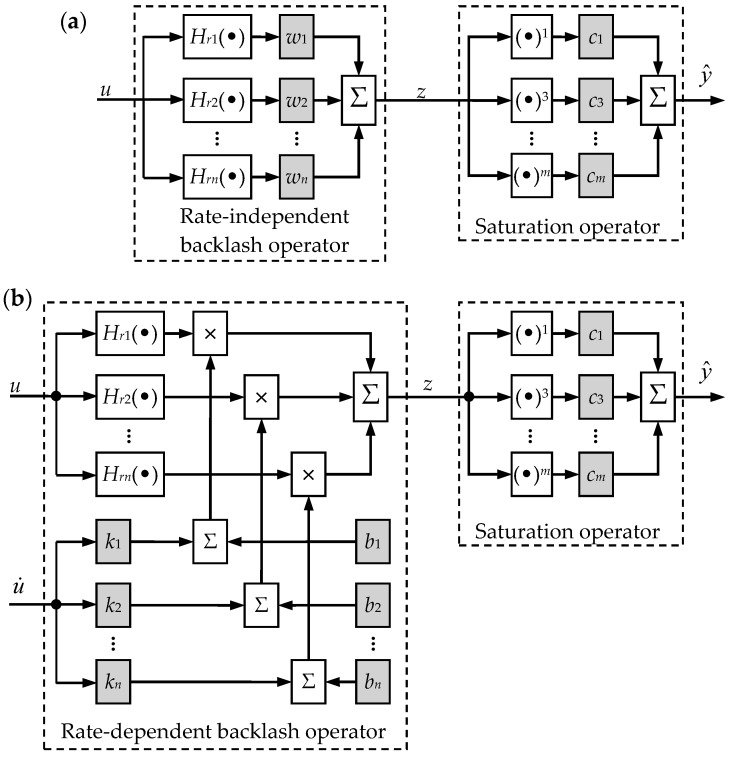
Schematic diagram of the modified Prandtl-Ishlinskii (PI) model: (**a**) rate-independent hysteresis model and (**b**) rate-dependent hysteresis model.

It is noted that the classical PI model shown in Equation (2) is rate-independent and symmetrical about its loop center. However, the measured hysteresis loops of PEAs are rate-dependent and asymmetric (saturation property). In order to improve the modeling accuracy, a modified PI model is proposed and schematically illustrated in Figure 2a, where an additional saturation operator is cascaded to the classical PI model. Following the notations in Equation (2), *H_rn_*(•) stands for the backlash operator in the classical PI model and *w_n_* is the weight vector in the backlash operator. A special polynomial operator with only odd powers in the following formulation is utilized as the saturation operator:

(3) $$\hat{y}(t)=S[z](t)=c_{1}z(t)+c_{3}z^{3}(t)+\cdots +c_{m}z^{m}(t),\;m=1,3,5,\cdots$$

where *S*[*z*](*t*) denotes the saturation operator, $\hat{y}(t)$ is the output, *c_i_* (*i* = 1, 3, 5, ..., *m*) is the coefficients of the polynomial, and *m* is the order of the polynomial. 

Unlike the common polynomials, all the even powers and the constant are totally eliminated to guarantee the axial symmetry of the saturation operator about the origin. Through literature review, one-sided dead-zone operator is another popular saturation operator [14,22]. However, this operator is piecewise in nature, even if the operator’s order increases. On the contrary, the coefficients of the polynomial are fewer but the curve of the polynomial is smooth, as verified in our previous work [20]. This helps to increase the modeling accuracy while decreasing the model complexity. In addition, more complex saturation property is possible by tuning the degree of the polynomial. 

Substituting Equation (2) into Equation (3), the rate-independent hysteresis model is written as:

(4) $$\hat{y}(t)=S\left[w^{T}\cdot H_{r}(u)\right](t)$$

#### 2.2.3. Modeling of the Rate-Dependence Property

The shape of the PEA’s hysteresis loop varies with different control inputs, e.g., the hysteresis loop become thicker if the rate or the frequency of the input increases. For the modeling of the rate-dependence, it has been verified that linearly tuning the weight vector of the modified PI model according to the input rate can significantly improve the modeling accuracy. The schematic diagram of the rate-dependent hysteresis model is given in Figure 2b. The linear relationship can be expressed in the following equation:

(5) $$w=k\cdot \dot{u}(t)+b$$

where **k** = [*k*_1_, *k*_2_, ..., *k_n_*]*^T^*, **b** = [*b*_1_, *b*_2_, ..., *b_n_*]*^T^*, *k_i_* and *b_i_* (*i* = 1, 2, ..., *n*) are the slop and offset vectors, respectively, and $\dot{u}(t)$ is the input rate.

Substituting Equations (2) and (5) into Equation (3), the rate-dependent hysteresis model is obtained:

(6) $$Y(t)=S\left\{{\left[k\cdot \dot{u}+b\right]}^{T}\cdot H_{r}(u)\right\}(t)$$

A similar modeling approach using a third order polynomial as the saturation operator was also proposed in [23], where the classical PI model and the saturation operator are connected in parallel. It is also noted that the hysteresis model developed in [23] is rate-independent. In our approach, the saturation operator is cascaded to the classical PI model in series, and the order of the polynomial can be tuned for more complex hysteresis. More importantly, the hysteresis model defined in Equation (6) is rate-dependent, and thus the modeling accuracy can be guaranteed.

### 2.3. Full Parameter Identification

The parameters that need to be identified include the threshold and weight vectors, and the coefficients of the saturation operator. The threshold vector is very important in the identification. Higher modeling accuracy can be achieved if a higher order of backlash operators and fine spacing are selected. However, higher order will increase the model complexity, and the computation time will also increase significantly, causing severe problems in real-time applications. A trade-off has to be made between the modeling accuracy and the system complexity. One practical solution is assigning fine spacing at low threshold values while assigning coarse spacing at larger threshold values. As a result, a threshold vector with 10th order or above is adequate to achieve satisfactory results. However, through literature review, this non-uniform spacing is typically assigned manually through a laborious trial and error process, and thus the prior experience on hysteresis modeling is highly demanded. This significantly affects the applicability of the PI-based approaches.

In our previous work [20], the threshold vector is manually assigned, and the saturation and backlash operators are identified separately for a shorter computation time. Thus, two sets of experimental data are required in the identification. In this paper, a highly efficient full parameter identification approach is proposed to identify all the parameters through only one set of experimental data. The practitioner only has to find out the input range of the system from the manual. No other prior experience on the PEA’s hysteresis or post processing, such as curve-fitting, is required. This guarantees ease of use and high efficiency, and thus signifies progress from our previous approach [20].

#### 2.3.1. Error Functions for Parameter Identification

When the PEA moves very slowly, e.g., tracking a trajectory below 1 Hz or following the trajectory of a master operator, the rate-dependence is negligible, resulting in a rate-independence (static) hysteresis. In this case, the rate-independent model defined in Equation (4) is sufficient to predict the output of the PEA. All the parameters in Equation (4) can be identified by comparing the model output with the measured hysteresis and minimizing the following error function:

(7) $$E\left[\hat{y},y\right](r,w,c,t)=\hat{y}(t)-y(t)=S\left[w^{T}\cdot H_{r}\right](t)-y(t)$$

where **r** = [*r*_1_, *r*_2_, ..., *r_n_*]*^T^* is the threshold vector.

If the PEA moves fast, the rate-dependence will become very obvious. In this case, the rate-dependent model defined in Equation (6) should be used to predict the output of the PEA. Similarly, all the parameters in Equation (6) can be identified by comparing the model output with the measured hysteresis and minimizing the other error function:

(8) $$E\left[\hat{y},y\right](r,k,b,c,t)=\hat{y}(t)-y(t)=S\left\{{\left[k\cdot \dot{u}+b\right]}^{T}\cdot H_{r}\right\}(t)-y(t)$$

In the parameter identification process, the method of least squares is adopted to minimize the error functions defined in Equations (7) and (8). The parameter identification is implemented in the environment of MATLAB (Version R2014a, MathWorks, Natick, MA, USA) and the function *lsqcurvefit* is selected. 

#### 2.3.2. Input Signals for Parameter Identification

For the rate-dependent hysteresis identification, it is straightforward to excite the system using control signals at different constant input rates (e.g., saw tooth signals with different slopes) and to identify the parameters in each case separately, as proposed in the research work of Ang et al. [14]. Subsequently, an additional curve fitting is conducted in order to obtain a general model that covers a certain range of input rates. This process is laborious as one has to make many measurements so as to obtain adequate data for the identification. Further, as saw tooth signals are not consistent, the modal vibrations of the PEA-driven system are likely to be excited by the high frequency components of the saw tooth signal.

Alternately, it is possible to identify all the parameters through only one set of experimental data if the input rate of the control signal is not constant but spans a certain range. Based on our previous work, the superimposition of multiple sinusoidal signals at different frequencies in the following form is a better alternative:

(9) $$u(t)=\sum_{N}A_{i}\sin\left(2\pi f_{i}t-\frac{\pi }{2}\right),\;N\geq 2$$

where *N* is the number of the sinusoidal signals, *A_i_* and *f_i_* are the magnitude and frequency of the sinusoidal signal, respectively.

The superimposition of multiple sinusoidal signals is superior to the saw tooth signal in that: (1) the input rate can span a wide range through a careful selection of the sinusoidal signals, and (2) the signal is consistent and the modal vibration can be avoided. 

#### 2.3.3. Non-Uniform Initialization of the Threshold Vector

As previously stated in this paper, non-uniform spacing of the threshold vector can achieve better modeling accuracy. As a result, during the parameter identification process, the threshold vector is initialized using a cubic relationship to guarantee fine spacing at small threshold values and coarse spacing at larger threshold values:

(10) $$r_{i}={\left(\frac{i}{n}\right)}^{3}\cdot \frac{U}{2},\;i=1,2,...,n$$

where *U* is the upper bound of the input signal.

## 3. Results

### 3.1. Rate-Independent Hysteresis Identification

**Figure 3:**
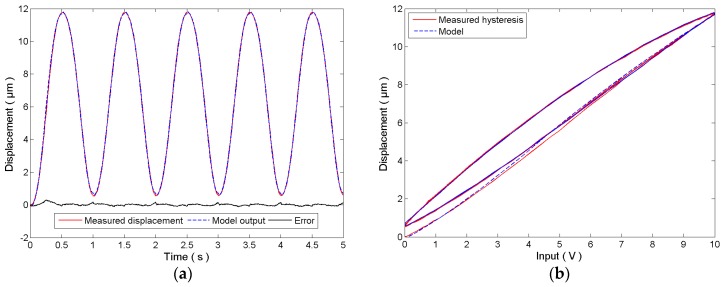
Identification of the rate-independent hysteresis model using a 1 Hz sinusoidal signal: (**a**) time plot and (**b**) hysteresis plot.

For the identification of the rate-independent hysteresis, a 10 Vp-p, 1 Hz sinusoidal signal is adopted as the control input to the system. The measured displacement of the PEA and the model output of the rate-independent hysteresis model in Equation (4) are plotted in Figure 3. It can be observed that the identified hysteresis model agrees with the measured hysteresis of the PEA. The modeling error is 5.287 ± 62.41 nm. The identified parameters are given below:

(11) $$\begin{array}{l} r={\left[0,\;4.933e^{-3},\;0.02294,\;0.1830,\;0.4944,\;1.109,\;1.839,\;2.617,\;3.779,\;4.922\right]}^{T}\\ w={\left[-2.31,\;2.137,\;0.35,\;9.3e^{-3},\;0.02532,\;0.02235,\;0.01895,\;0.01826,\;0.02765,\;-0.01929\right]}^{T}\\ c={\left[-8.794e^{-4},\;0,\;-0.05066,\;0,\;4.818\right]}^{T} \end{array}$$

### 3.2. Rate-Dependent Hysteresis Identification

**Figure 4:**
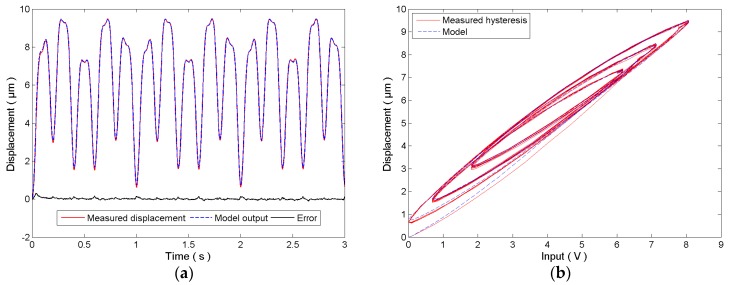
Identification of the rate-dependent hysteresis model using the superimposition of two sinusoidal signals: (**a**) time plot and (**b**) hysteresis plot.

In the identification of the rate-dependent hysteresis, the superimposition of two sinusoidal signals is chosen where *A*_1_ = 2, *f*_1_ = 10, and *A*_2_ = 3, *f*_2_ = 5, respectively. This control signal is then exerted on the PEA. The parameters of the rate-dependent hysteresis model are identified according to the error function in Equation (8). The measured displacement of the PEA and the model output are plotted in Figure 4. Similar to the results in the rate-independence case, the identified model follows the measured hysteresis of the PEA well with a modeling error of 3.526 ± 45.55 nm. The identified parameters are given below:

(12) $$\begin{array}{l} r={\left[0,1.772e^{-3},0.03242,0.08513,0.1940,0.8016,1.356,2.104,3.055,3.438\right]}^{T}\\ k={\left[-0.1041,0.1075,-3.254e^{-3},-7.082e^{-6},-1.974e^{-4},-9.755e^{-6},1.232e^{-6},-2.045e^{-5},4.844e^{-5},-5.606e^{-5}\right]}^{T}\\ b={\left[-0.5510,0.6097,-0.01653,0.01098,1.262e^{-3},4.928e^{-3},4.950e^{-3},6.183e^{-3},6.393e^{-3},-3.426e^{-4}\right]}^{T}\\ c={\left[0.5682,0,-3.391,0,17.54\right]}^{T} \end{array}$$

### 3.3. Verifications of the Rate-Independent and Rate-Dependent Models

**Figure 5:**
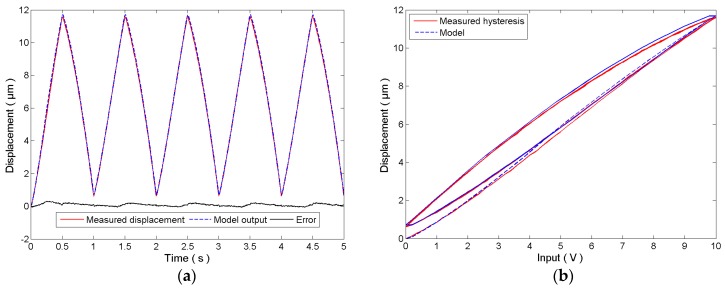
Verification of the rate-independent hysteresis model using another 1 Hz triangular signal: (**a**) time plot and (**b**) hysteresis plot.

The identified rate-independent hysteresis model is verified using a 10 Vp-p, 1 Hz triangle signal. The experimental results are given in Figure 5. It is observed that the model output follows the measured displacement well and the estimation error is measured to be 79.56 ± 77.6 nm, on the same order of the noise level of the system. Therefore, the rate-independent is applicable for slow trajectories, e.g., below 1 Hz. Taking the relatively simple structure into consideration, the rate-independent hysteresis model is a better choice if the system moves slowly.

**Figure 6:**
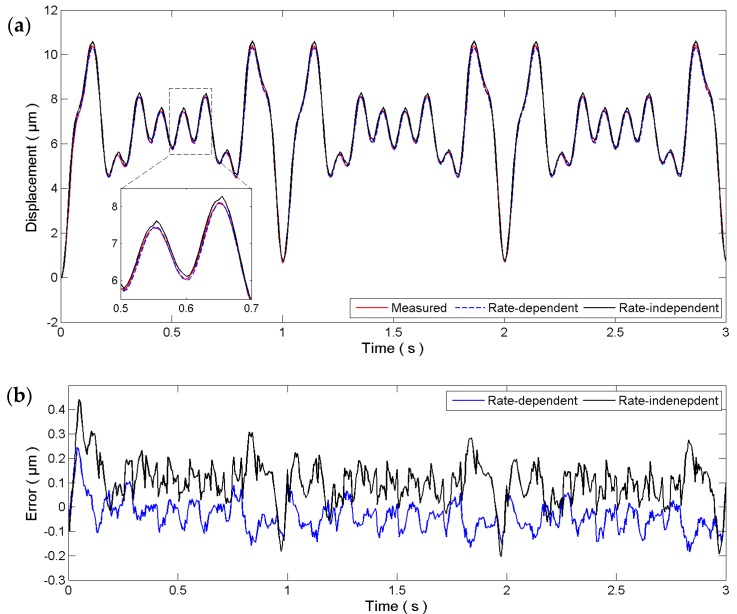
Verification of the identified hysteresis models using a superimposition of four sinusoidal signals: (**a**) time plot and (**b**) error plot.

For higher frequencies, both the identified rate-independent and rate-dependent hysteresis models are verified using an input signal consisting of four sinusoidal signals with *A*_1_ = 1, *f*_1_ = 10, *A*_2_ = 1, *f*_2_ = 7.5, *A*_3_ = 1, *f*_3_ = 5, *A*_4_ = 2, and *f*_4_ = 4. The measured displacement and the model outputs are given in Figure 6a. It can be observed that both the rate-independent and rate-dependent models can follow the measured displacements well. The estimation errors are measured to be 88.10 ± 86.29 nm and −48.77 ± 57.22 nm for the rate-independent and rate-dependent models, respectively. The estimation error of the rate-independent model is slightly higher than its modeling error. This is reasonable as the rate-independent model is identified using a 1 Hz sinusoidal signal and focuses mainly on low-frequency signals. The high-frequency components of the superimposed signal will definitely affect the accuracy of the rate-independent model. On the contrary, the estimation error of the rate-dependent model is on the same order of the modeling error. The error plot in Figure 6b clearly shows that the rate-dependent model achieves better performance than the rate-independent model for fast trajectories.

**Figure 7:**
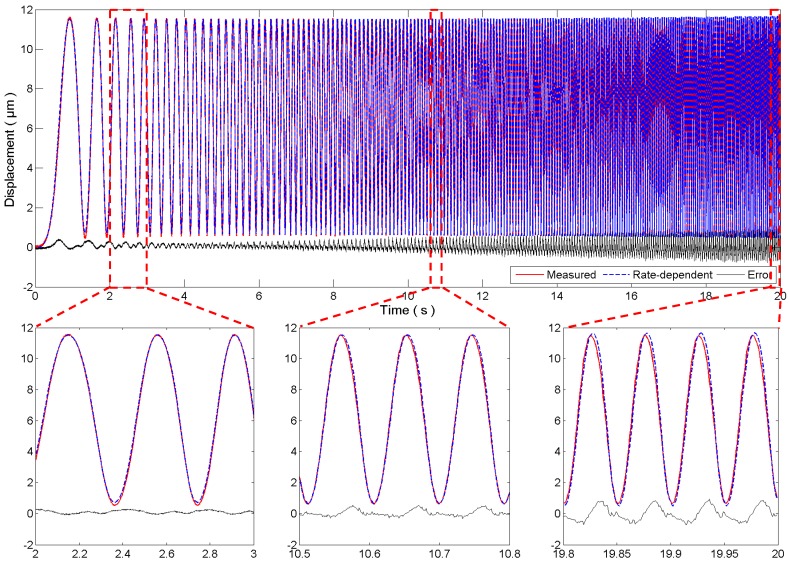
Verification of the rate-dependent model using a 0.1–20 Hz swept sinusoidal signal.

Because the superimposed sinusoidal signal in Figure 6 only consists of four frequencies, experiments are further conducted to test the performance of the identified rate-dependent model using a 0.1–20 Hz swept sinusoidal signal. Compared with the superimposed sinusoidal signal, the swept sinusoidal signal is also smooth but it contains all the frequency components between 0.1 Hz and 20 Hz, and thus the overall performance of the rate-dependent model over a wider frequency range can be examined. The experimental results are given in Figure 7. It is observed that the rate-dependent model can still follow the measured displacement well, as observed in the zoomed-in insets in Figure 7. However, since the maximum frequency component in the identification is only 10 Hz, the modeling accuracy for higher frequencies is not guaranteed. Experimental results in Figure 7 show that the estimation error will become larger for higher frequencies. The maximum estimation error in the last one second is 94.11 nm, corresponding to 8.25% of the measured displacement. Therefore, the identified rate-dependent model is applicable for fast trajectories with a frequency range of 20 Hz.

## 4. Discussion

The hysteresis modeling and compensation has become an important issue in the motion control of PEAs. The rate-dependence and saturation (asymmetry) phenomena are observed in the measured hysteresis curves of PEAs. In this paper, the PI model is selected to build the hysteresis model. Two important modifications are made to the classical PI model: (1) the weights of the backlash operators are dynamically updated according to the change in the input rate so as to account for the rate-dependence, and (2) a polynomial operator with only odd powers is cascaded to the backlash operators to adjust the shape of the hysteresis loop to model the saturation property. 

The parameters that need to be identified include the threshold and weight vectors of the backlash operators and the coefficients of the polynomial operator. The efficiency of the conventional parameter identification approach is low because of the huge amount of experimental data and the time-consuming post processes, such as the curve fitting. Further, the sudden change in the input signal might excite higher order modal vibrations of the system during the experiment. Another problem in the parameter identification is the manual intervention. For instance, the threshold vector is generally manually assigned to guarantee fine spacing at small values and coarse spacing at larger values. This task requires rich experience on the hysteresis modeling of PEAs, and thus is not practical for beginners or common practitioners with limited experience. Therefore, it is necessary to simplify the parameter identification to achieve minimal manual intervention. 

A full parameter identification approach is proposed in this paper. The basic idea is to choose a special input signal covering a wide range of input rates so that the response of the PEA to different input rates can be obtained in one single measurement. The superimposition of multiple sinusoidal signals with different frequencies is an ideal input signal as it covers a wide range of input rates. More importantly, the input signal is smooth and will not excite the higher modal vibration of the system. Subsequently, the method of least squares is utilized to identify all the parameters automatically without any manual intervention. This methodology is superior in that prior experience on hysteresis modeling is not required any more, thus guaranteeing ease of use even for the beginner.

The effectiveness of the proposed methodology is verified on a PEA-driven flexure mechanism. Both rate-independent and rate-dependent hysteresis identifications are conducted. Experimental results show that the rate-independent model is adequate to describe the motion of the PEA if the PEA works in slowly moving scenarios, such as following the trajectory of a master operator. For the rate-dependent hysteresis model, experimental results show that the modeling accuracy is high in the frequency range of 20 Hz.
